# Serum Stability and Physicochemical Characterization of a Novel Amphipathic Peptide C6M1 for SiRNA Delivery

**DOI:** 10.1371/journal.pone.0097797

**Published:** 2014-05-15

**Authors:** Mousa Jafari, Wen Xu, Ran Pan, Chad M. Sweeting, Desiree Nedra Karunaratne, Pu Chen

**Affiliations:** 1 Department of Chemical Engineering, University of Waterloo, Waterloo, Ontario, Canada; 2 Waterloo Institute for Nanotechnology, University of Waterloo, Waterloo, Ontario, Canada; 3 Department of Chemistry, University of Peradeniya, Peradeniya, Sri Lanka; University of Helsinki, Finland

## Abstract

The efficient delivery of nucleic acids as therapeutic agents is a major challenge in gene therapy. Peptides have recently emerged as a novel carrier for delivery of drugs and genes. C6M1 is a designed amphipathic peptide with the ability to form stable complexes with short interfering RNA (siRNA). The peptide showed a combination of random coil and helical structure in water but mainly adopted a helical conformation in the presence of anions or siRNA. Revealed by dynamic light scattering (DLS) and microscopy techniques, the interaction of C6M1 and siRNA in water and HEPES led to complexes of ∼70 and ∼155 nm in size, respectively, but showed aggregates as large as ∼500 nm in PBS. The time-dependent aggregation of the complex in PBS was studied by DLS and fluorescence spectroscopy. At molar ratio of 15∶1, C6M1 was able to completely encapsulate siRNA; however, higher molar ratios were required to obtain stable complexes. Naked siRNA was completely degraded in 4 h in the solution of 50% serum; however C6M1 protected siRNA against serum RNase over the period of 24 h. Western blotting experiment showed ∼72% decrease in GAPDH protein level of the cells treated with C6M1-siRNA complexes while no significant knockdown was observed for the cells treated with naked siRNA.

## Introduction

Over the past two decades, major advances have been made in the field of gene therapy. Since its discovery, RNA interference (RNAi) has provided new perspectives in developing novel nucleic acid (NA)-based therapeutics [1]–[3]. However, their development has been restricted by their poor stability and cellular uptake. NAs are vulnerable to enzymatic degradation in physiological environment, declining their potency, and lack the ability to cross impermeable barriers of biological membranes. For the clinical advancement of RNAi, the design and development of safe and effective delivery systems is vital. Several viral and non-viral delivery systems, including lipids [4], [5], polymers [6], [7], and peptides [8], [9] have been engineered and developed to obtain desired capabilities to overcome the cellular delivery barriers.

Cell penetrating peptides (CPPs) are short positively-charged peptides, usually less than 30 amino acids, with the ability to cross the cellular plasma membrane. CPPs have been reported to mediate the delivery of a large panel of cargos including siRNA, plasmid DNA, protein, and liposome *in vitro* and *in vivo* [10]–[12].Two different strategies are mainly applied to form peptide-cargo conjugates: either peptides are covalently attached to the cargo, or they interact through non-covalent, mainly electrostatic, interactions to form complexes. Taking the opposite charges of CPPs and NAs into account, the non-covalent approach has been mostly applied for the formulation of peptide-NA complexes.

Considering the amphiphilic nature of the cell membrane, the majority of protein-derived and designed CPPs are amphipathic. This feature facilitates the interaction of peptide with charged phospholipids or proteoglycans on the surface of the cell membrane and hydrophobic core of the bilayer. It also enables peptides to interact with both hydrophilic and hydrophobic drugs. The amphiphilicity of the peptides may evolve from their primary structure, e.g., MPG [13], or secondary structure, e.g., CADY [14], and Penetratin [15]. In Primary amphipathic peptides, the hydrophilic and hydrophobic moieties are located in the two opposite ends of the peptide sequence while, secondary amphipathic peptides are required to adopt a helical structure in order to organize hydrophilic and hydrophobic moieties at opposite sides of the helix [16], [17].

In general, siRNA, which has a short structure compared to DNA, lack stability in the presence of nucleases. This is a real challenge for systematic administration of NA-based drugs besides their poor cellular uptake. Several attempts have been made to stabilize siRNA under physiological conditions. Chemical modifications in the nucleobases, sugars, and the phosphate ester backbone of siRNA have been shown to increase its nuclease resistance without interfering with its biological activities [18]–[20]. Polymerization of siRNA has been also demonestared higher stability as it make so-called stick siRNA look like a gene [21]. Alternatively polycations have been widely reported to protect NAs against enzymatic degradation due to their opposite charge and ability to form stable complexes through covalent and/or non-covalent interactions.

As the cellular uptake of the cargo-drug complexes is highly influenced by the size and charge of the complex [22], choosing an appropriate solvent or buffer to prepare the solution is of high importance. The pH and anionic strength of the buffer can greatly affect the size and surface charge and eventually the stability of the complex. Phosphate buffered saline (PBS) has been widely used in *in vitro* and *in vivo* studies as the osmolarity and ion concentrations of this buffer match those of the human body. However, consideration should be taken into account as high concentration of anions in this buffer can contribute to the aggregation of cationic carriers even before the formation of the complex.

In the present work we characterize the interaction of a designed amphipathic peptide, C6M1, with siRNA, using several spectroscopic and microscopic techniques. The change in size and charge of the C6M1-siRNA complexes in different media is discussed. The stability of C6M1-siRNA complexes in the presence of heparin and serum is examined using gel electrophoresis. The effect of interaction with siRNA and anions on the secondary structure of C6M1 is also explained.

## Materials and Methods

### Materials

N-terminal acetylated and C-terminal amidated C6M1 peptide (MW = 2689.4 g/mol, purity>98%) was purchased from CanPeptide, Inc. (Quebec, Canada). Glyceraldehyde 3-phosphate dehydrogenase (GAPDH) siRNA (AM4631) was purchased from Ambion (Austin, USA). All chemicals for buffer preparations were obtained from Sigma-Aldrich (Oakville, ON, Canada) and used as received.

### Formulation of peptide-siRNA complexes

The peptide stock solution (1 mM) was prepared by dissolving peptide powder in RNase free water. The solution was then vortexed for 10 seconds and sonicated for 10 minutes in a tabletop ultrasonic cleaner (Branson, model 2510, USA). The siRNA stock solution (50 µM) was also prepared by dissolving peptide powder in RNase free water. Peptide-siRNA complexes were formed by adding peptide solution into siRNA in proportion according to the designed experiment and diluting in RNase free water, HEPES (6 mM, pH = 7.4), or phosphate buffered saline, PBS (pH 7.4, 137 mM NaCl, 2.7 mM KCl, 10 mM Na_2_HPO_4_, 1.8 mM NaH_2_PO_4_), to achieve the final concentrations. The complexes were incubated for 20 minutes at room temperature before each experiment, unless specified otherwise.

### Dynamic Light Scattering (DLS) and Zeta potential

The size of the peptide-siRNA complexes was measured on a Zetasizer Nano ZS (Malvern Instruments, U.K.) equipped with a 4 mW He-Ne laser operating at 633 nm. Samples at molar ratios of 1∶1 to 60∶1 with final siRNA concentration of 100 nM were prepared as mentioned above. A quartz microcell (45 µL) with a 3 mm light path was used and the scattered light intensities were collected at an angle of 173°. Clear disposable zeta cells were used for Zeta potential measurements. The size distribution and zeta potential values were acquired using the multimodal algorithm CONTIN, Dispersion Technology Software 5.0. Three independent measurements were performed for each sample 20 min after sample preparation at 25°C.

### Transmission electron microscopy (TEM)

5 µl samples of peptide/siRNA complexes at siRNA concentration of 200 nM and molar ratio of 30∶1 in water, HEPES, and PBS were deposited onto 400 mesh Formva coated copper grids (Canemco-Marivac, Canada) for 10 minutes. The excess was blotted with a filter paper. The grids were then washed by plunging into an RNase free water bath, followed by drying overnight. The samples were stained with 2% uranyl acetate solution (Electron Microscopy Sciences) and analyzed by TEM (Philips CM10 TEM).

### Fluorescence spectroscopy

Since C6M1 has four tryptophan residues as intrinsic fluorescent probes, fluorescence spectroscopy was applied to characterize the interaction between siRNA and peptide. The peptide fluorescence was acquired on a Photon Technology International spectrofluorometer (Type LS-100, London, Canada) with a pulsed xenon lamp as the light source. Samples (80 µl) were transferred to a quartz cell (1 cm×1 cm) and excited at 280 nm and spectra were collected in the range of 300–500 nm. The standard fluorescence intensity I_s_ was obtained by taking the average of the fluorescence of peptide only sample (160 µM) from 480 to 500 nm.

### Circular Dichroism (CD) spectroscopy

C6M1-siRNA complexes in water or HEPES-buffered saline (HBS: 6 mM HEPES, 150 mM NaCl) at molar ratios of 10∶1, 20∶1, and 40∶1 were prepared at fixed C6M1 concentration of 80 µM and varying concentration of siRNA. Spectra from 250 to 190 nm with spectral resolution and pitch of 1 nm and scan speed of 200 nm/min were recorded with a J-810 spectropolarimeter (Jasco, USA). Samples were transferred into 1 mm long quartz cells and maintained at 25°C. Spectra shown are the average of three replicates. The raw CD ellipticity (in millidegrees) was converted to residue molar ellipticity (deg.cm^2^.dmol^−1^.residue^−1^): θ = θ_raw_/(10×C×N×l), where θ_raw_ is the ellipticity in millidegrees, C is the peptide concentration (mol/L), l is the optical path length of the cell (cm) and N is the number of residues. The secondary structure composition of the peptide was estimated from CD spectra using K2D3 program [23].

### Gel electrophoresis

To study the ability of C6M1 to co-assemble with siRNA, agarose gel electrophoresis was carried out at 50 V for 60 min in TBE buffer (4.45 mM Tris–base, 1 mM sodium EDTA, 4.45 mM boric acid, pH 8.3). C6M1 and siRNA were mixed at different molar ratios ranging from 1∶1 to 40∶1 and incubated at 37°C for 20 min, to form complexes. Samples were analyzed on a 0.8% wt/vol agarose gel, stained with 0.5 µg/ml ethidium bromide and revealed by UV illumination.

To evaluate the stability of the complexes at different molar ratios, the heparin competition assay was performed. Different amounts of heparin corresponding to final concentrations from 0.5 to 10 µg heparin per 10 µl of the complex were added to C6M1/siRNA complexes at molar ratios of 15∶1, 40∶1, 60∶1, and 80∶1. Ten microliters of each sample, corresponding to 50 pmol of siRNA, was then analyzed by electrophoresis on agarose gel (1.2% wt/vol) stained with ethidium bromide.

### Stability of naked siRNA and complexes in the presence of serum

The ability of C6M1 in protecting siRNA against degradation by serum components was studied by agarose gel electrophoresis. C6M1-siRNA complexes at molar ratio of 30∶1 were incubated with equal volume of fetal bovine serum (FBS) (final concentration of 50% v/v) at 37°C. The inactivated serum, treated with 0.5M EDTA, was used as a control. 20 µl aliquots were taken at 30 min, 2 h, 4 h, 6 h, 18 h and 24 h. 1 µl of 0.5 M EDTA was immediately added to stop the degradation. After the addition of 1% heparin to displace siRNA from the complex, 10 µl of each sample, corresponding to 50 pmol of siRNA was analyzed by 0.8% agarose gel electrophoresis.

### C6M1-mediated siRNA knock down analysis by Western blotting

Chinese hamster ovary cells, CHO-K1 (ATCC -CCL-61), were cultured in 12-well cell culture plates at a concentration of 80000 cell/ml to reach ∼60% confluency the next day. 24 h later, the medium was replaced with Opti-MEM. The complexes of C6M1 with GAPDH siRNA or scrambled (negative control) siRNA were first prepared in water then introduced to PBS as the osmolarity and ion concentrations of this buffer match those of the human body. The complexes were then diluted in Opti-MEM to final siRNA concentration of 50 nM or a range of siRNA concentration from 5 to 100 nM at molar ratio of 30∶1 and incubated in 37°C for 20 min. The complexes or naked siRNA were then added to the cells and incubated at 37°C humidified atmosphere containing 5% CO2. 3 hours later, growth medium with 20% FBS was added. 24 h post-treatment, the cells were washed with PBS. Cells were detached by adding trypsin 48 hours after transfection, incubated with ice-cold lysis buffer 50 mM Tris-base, 150 mM NaCl, pH 8.0, 1% Triton X-100) containing Protease Inhibitor Cocktail (Cell Signaling Tech.) for 20 min, mixed every 5 min and then centrifuged at 4°C for 10 min at 13000 g. The supernatant were collected and total protein concentration was measured using BCA protein assay kit (Pierce). 15 mg cell extract proteins were separated by 12% SDS-PAGE and transferred onto a nitrocellulose membrane, blocked with TBS containing 5% dried skimmed milk for 1 h, followed by overnight incubation at 4°C with mouse anti-β-actin (AM4302, Ambion) and mouse anti-GAPDH (AM4300, Ambion). After washes in 0.05% Tween in PBS, the membrane was incubated with anti-mouse-HRP secondary antibody (Sigma-Aldrich). The blots were exposed by ECL Plus substrate and developed on X-Ray film (Fisher Scientific).

## Results and Discussion

### Peptide structure

**Figure 1:**
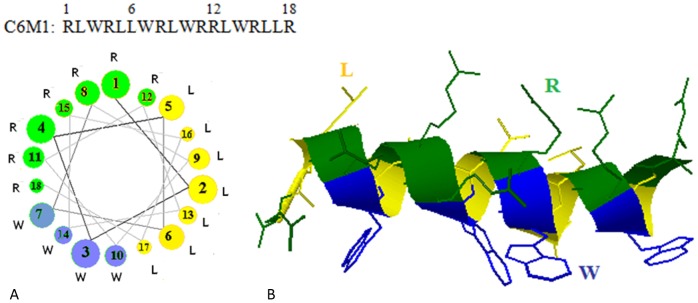
Helical structure and helical wheels representation of C6M1. A) A downward cross-sectional view of the helix axis is shown. The axis of the alpha helix is orthogonal to the paper plane. The bigger the circle is, the closer is the location of the residue to the upper end, when viewing from the top, B) In helical structure, same amino acids (side chains) face the same side of the helix. The schematics was generated using RaptorX web server [24]. R (green), L (yellow), and W (blue) represent arginine, leucine and tryptophan residues, respectively.

Considering several factors, including peptide self-assembly, peptide-siRNA co-assembly, siRNA loading and protection capability, peptide C6M1 was designed [17]. **Figure 1** shows the sequence, helical structure, and helical wheel projection of C6M1. The distribution of amino acids in C6M1 sequence enables the appearance of the same residues on the same face of the helix, inducing the amphiphilicity to the peptide. This arrangement facilitates the interaction of the peptide with siRNA and cell membrane and mediates the internalization of the peptide and its associated complex.

It is generally believed that the hydrogen bonding between N-H and C = O groups in the backbone of the peptide is involved in the formation of the helical structure. However, the role of hydrophobic, hydrogen bonding, π-π stacking, and electrostatic interactions between side chains in stabilizing the helices has also been revealed [25], [26]. Considering the unique arrangement of amino acids in C6M1 helical structure, hydrophobic interaction between leucine residues, π-π stacking interaction between tryptophan residues, and hydrogen bonding between arginine residues are expected to stabilize the helical conformation of the peptide (Figure 1).

### Size and surface charge of the C6M1-siRNA complexes in different media

**Figure 2:**
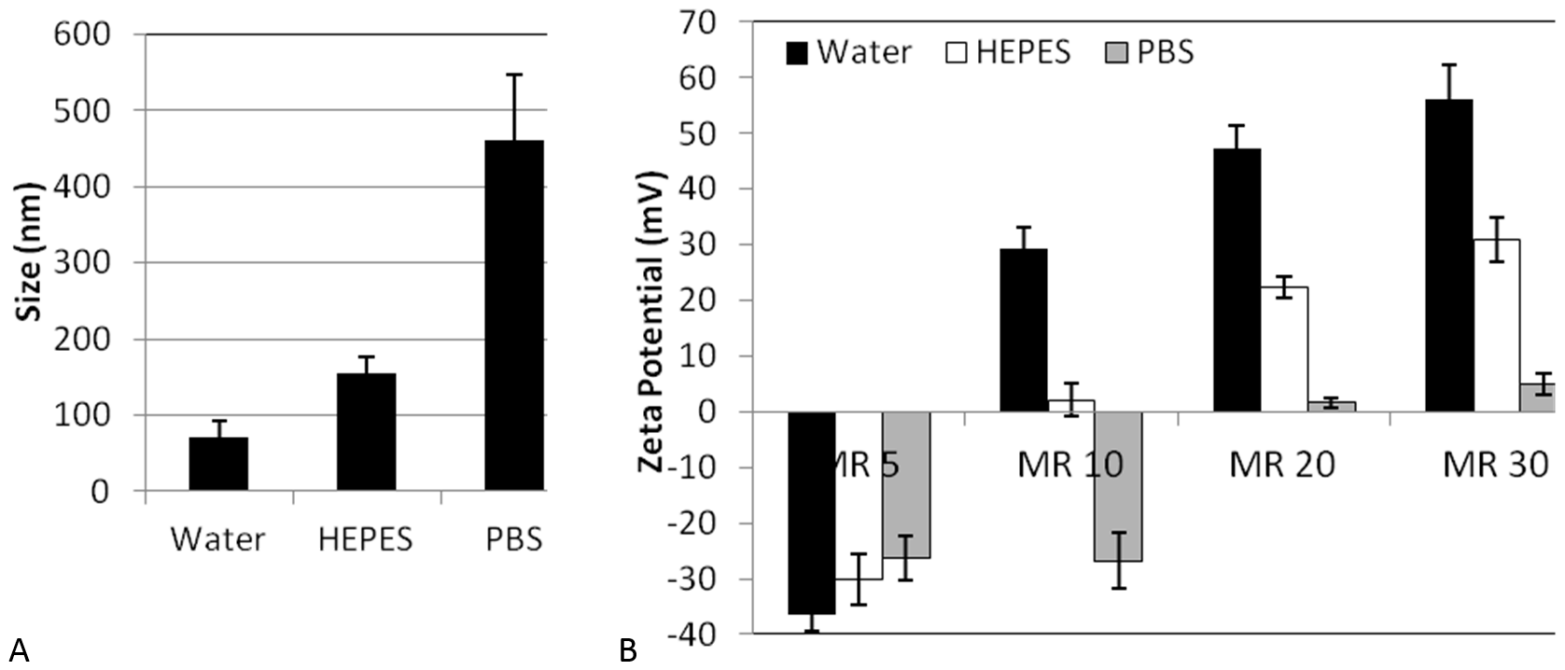
A) Size of the C6M1-siRNA complexes in water, HEPES, and PBS. B) Zeta potential of C6M1-siRNA complexes at different molar ratios in water (black bars), HEPES (white bars), and PBS (grey bars). Error bars represent standard deviation of triplicates. (MR =  C6M1:siRNA molar ratio)

The size of the C6M1-siRNA complexes at molar ratio of 30∶1 was measured by dynamic light scattering. The complexes were incubated for 20 min in water, HEPES, or PBS prior to size measurement. As shown in Figure 2A, complexes in water and HEPES buffer showed a size of ∼70 and ∼155 nm, respectively; however, the incubation in PBS led to the aggregation of the complex (∼460 nm). As the surface charge of the complex has been reported to affect its size, bio-distribution, and cellular uptake [27], zeta potential experiments were carried out for the complexes in water, HEPES, and PBS at molar ratios from 5∶1 to 30∶1. As shown in Figure 2B, complexes in all three media showed a negative zeta potential at MR of 5∶1, implying that the peptide molecules were not enough to neutralize all negatively-charged siRNA molecules. At MR of 10, complexes in water and HEPES showed positive zeta potential; while those in PBS possessed negative surface charge. This indicated that the ions in PBS may interfere with the electrostatic interaction between the peptide and siRNA, leading to mostly exposed siRNAs. Increasing the MR to 30∶1 led to zeta potential of +57, +31 and +5 mV in water, HEPES, and PBS, respectively. The zeta potential at higher molar ratios remained almost unchanged (not shown). The charge repulsion in the highly-charged complexes in water prevented their aggregation, keeping the size around 70 nm. HEPES, as a zwitterionic buffering agent, can maintain the physiological pH without significant contribution to the ionic strength of the solution and is a commonly-applied buffer in cell culture media. The low ionic strength of HEPES increased the size of the complex to ∼155 nm, considering the zeta potential of +31 mV. This change in the size of the complexes is of importance as it may affect the complex cellular uptake mechanism (unpublished data). In contrast, the high concentration of phosphate and chloride anions in PBS mainly neutralized the positive charge of the complex surface, as arginine residues on the surface of the complexes could act as phosphate and chloride binding sites [28]. This promoted the aggregation of the almost neutral particles mainly through hydrophobic interaction of leucine residues.

In a similar study, Hao et al. reported the dependency of size and charge of a cationic polymer-DNA complex on the nature of the media [29]. In their experiments, the complex had a high zeta potential and low particle size in water and 5% glucose solution. In contrast, PBS, DMEM or saline, lowered the zeta potential and mediated the aggregation of the particles. This is in accord with our observation and indicates that the ions in the medium could alter the size and charge of the complexes of cationic peptides/polymers with NAs.

**Figure 3:**
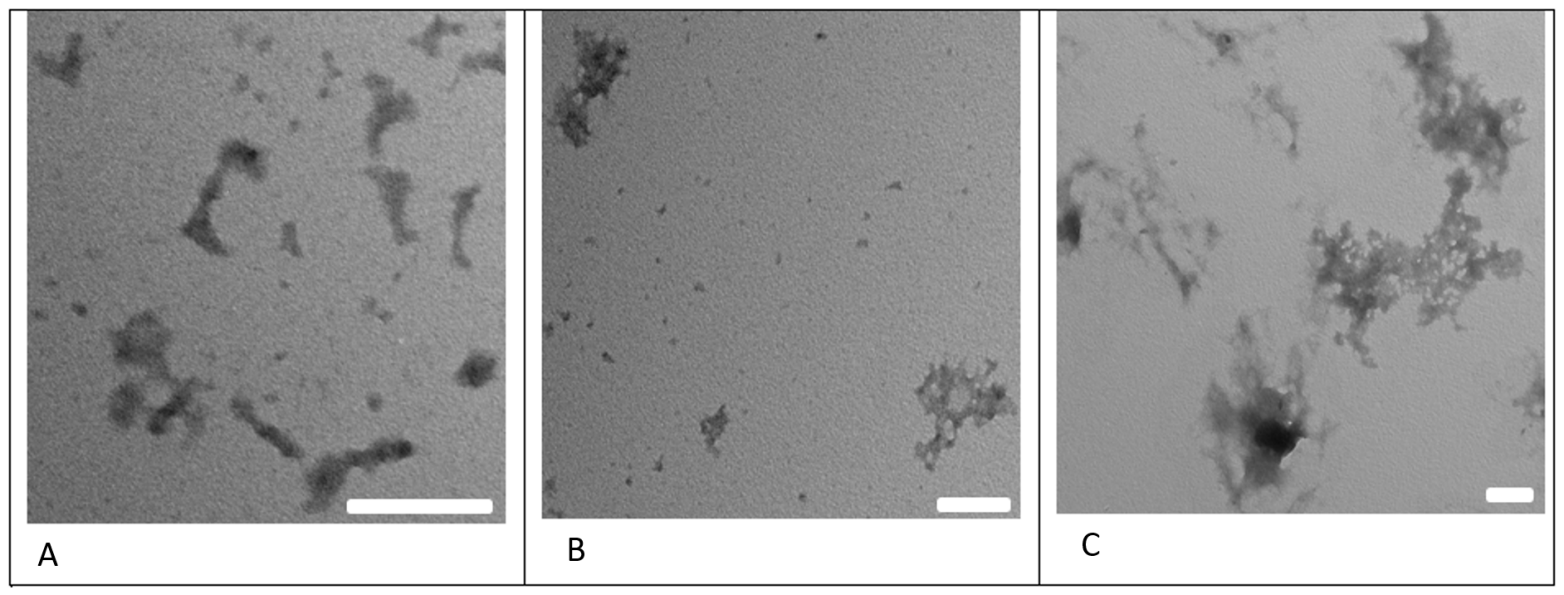
Transmission electron microscope images of C6M1-siRNA complexes (MR = 30∶1) in water (A), HEPES buffer (B), and PBS (C). Scale bars are 100

These findings were confirmed by TEM images. Figure 3 shows the TEM images of C6M1-siRNA complexes in water, HEPES and PBS. The sample in water and HEPES showed nanoparticles of irregular shape with size less than 100 and 200 nm, respectively; whereas, those in PBS showed aggregates of larger than 500 nm.

### Time-dependent aggregation of C6M1-siRNA in PBS

**Figure 4:**
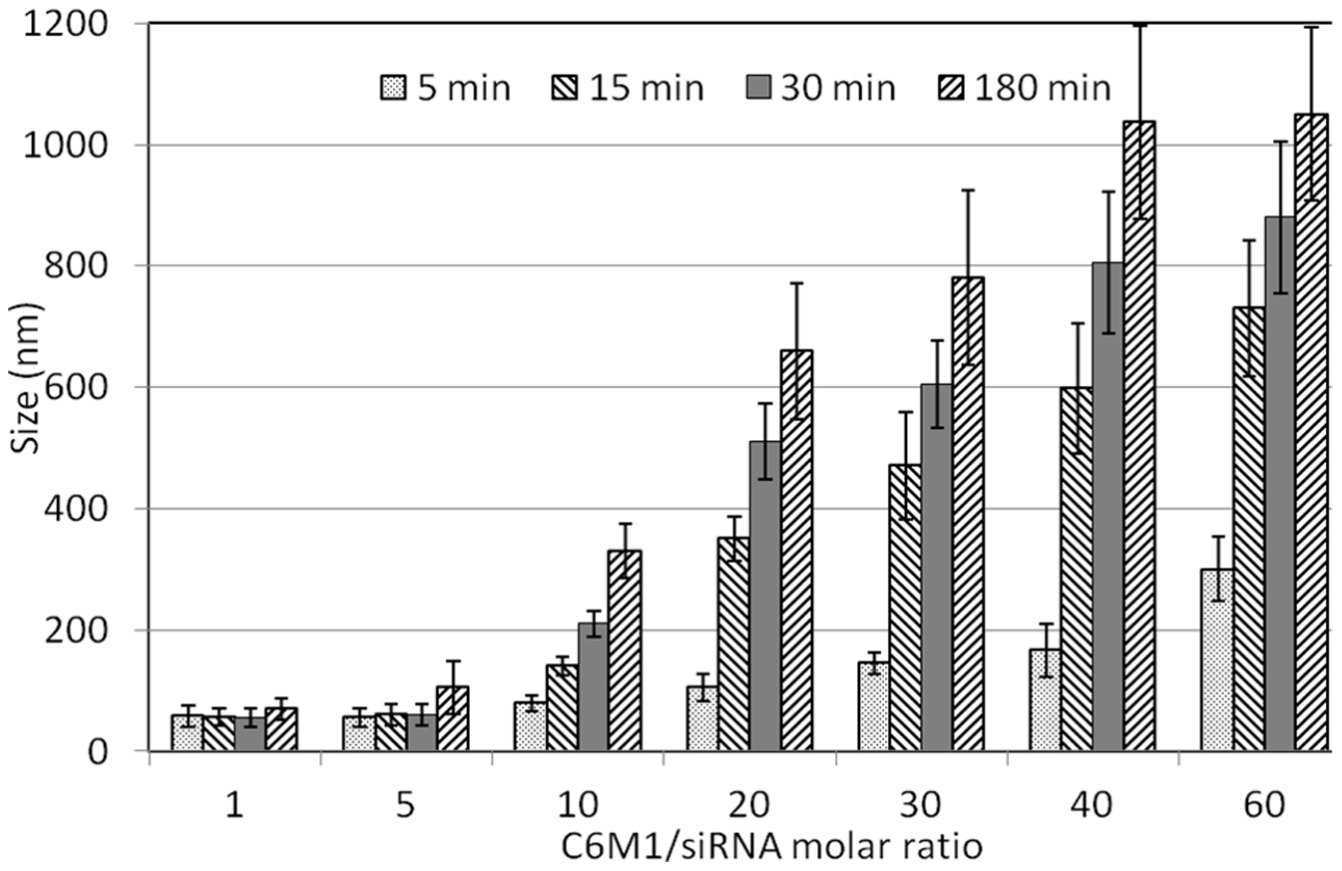
Size of C6M1/siRNA complexes at different molar ratios in PBS solution over time. Error bars represent standard deviation of three independent experiments.

The size of the C6M1-siRNA complex formed in PBS varied with time and the peptide:siRNA molar ratio (MR). As shown in Figure 4, the size of the complex remained unchanged at the MR of 1∶1. At the MR of 5∶1, the size of the complex changed from 50 to 120 nm over a time period of 3 h. When the ratio was increased to 20∶1, larger aggregates (∼500 nm) were observed in a short time of 30 min. Further investigation showed that the size of the complex could grow up to 1 µm at higher MRs (Figure 4). It should be noted that change in the size of the complexes was only observed in PBS and the size of the complexes in water and HEPES remained below 100 and 200 nm, respectively, even after 24 h incubation. These results show the importance of choosing suitable media especially during the formulation process, to avoid the aggregation or degradation of the complex which can greatly affect its functionality. Considering the buffering capabilities and “salt free” nature, HEPES was suggested as the solution for peptide-siRNA formulation.

**Figure 5:**
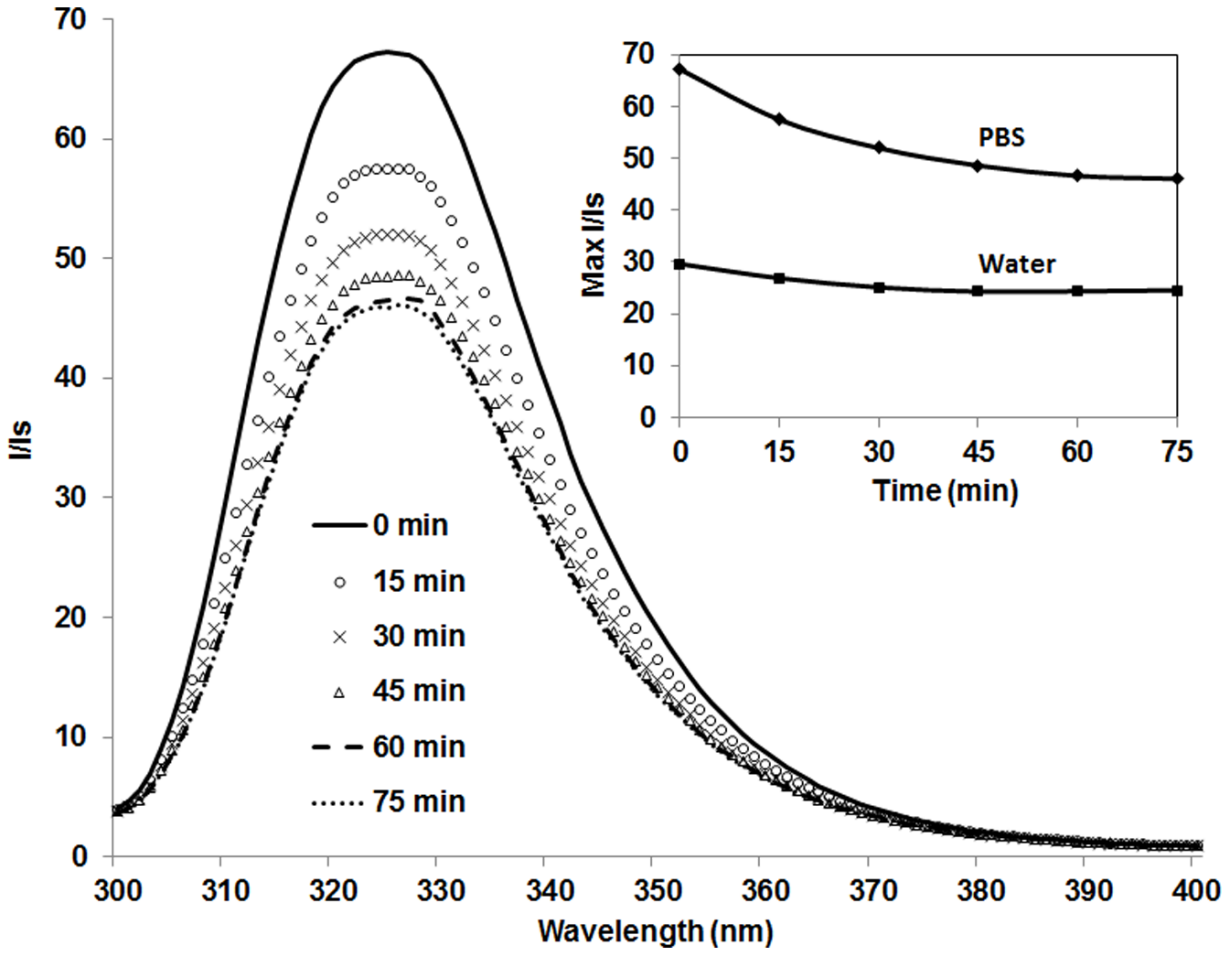
Change in fluorescence intensity of C6M1-siRNA complex (MR = 20∶1) over time in PBS. Inset Plot shows the change in maximum fluorescence intensity of the complex in PBS and Water over time.

Using tryptophan residues in C6M1 as internal fluorescent probes, the change in fluorescence spectra of C6M1-siRNA complexes at MR of 20∶1 was measured over a time period of 70 min. As tryptophan fluorescence is sensitive to the local environment, changes in the fluorescence emission spectra provide information on the conformation and aggregation of the peptide [30]. As shown in Figure 5, a decrease in the fluorescence intensity of the complex was observed over time until it reached a plateau at ∼60 min. This change in fluorescence intensity could only correlate to the change in the particle size as no other physicochemical parameter was changed during the experiment. Increasing the size of the complexes minimized the total surface area and number of tryptophan residues on the surface of the complexes, compared to smaller complexes, leading to decrease in the fluorescence intensity. The DLS experiment also revealed that there was no significant change in size of the complexes after ∼60 min (not shown). The change in the fluorescence of the complex in water was negligible since there was no significant aggregation of the complex (Figure 5, inset).

### Conformational Changes of C6M1 upon interacting with siRNA

**Figure 6:**
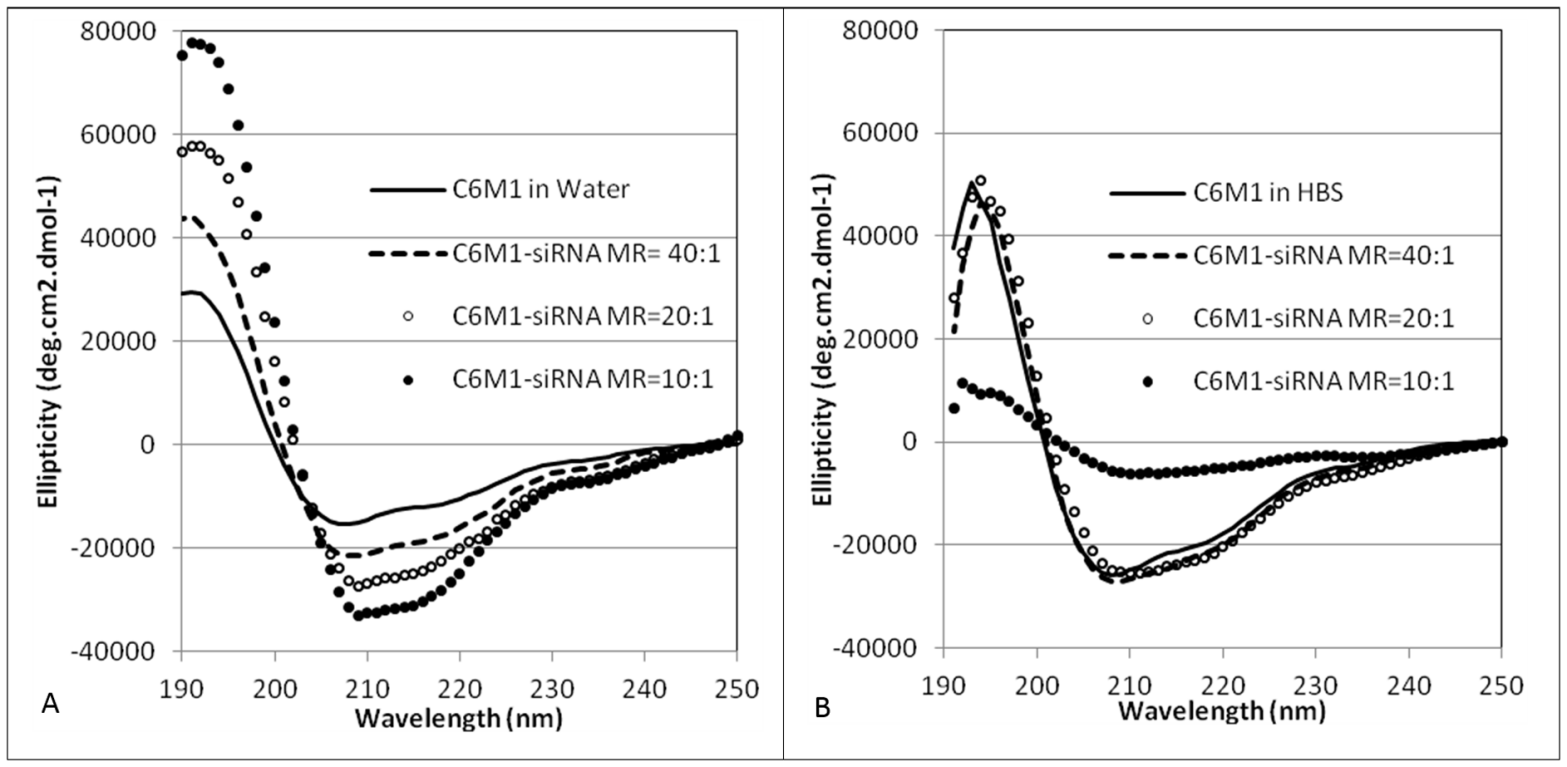
CD spectra of C6M1 peptide (80 µM) with varying amounts of siRNA in water (A) and in HBS (B). (MR =  C6M1:siRNA molar ratio).

The impact of siRNA on the secondary structure of C6M1 in water and HBS (6 mM HEPES, 150 mM NaCl) was evaluated by CD spectroscopy. As shown in Figure 6A and Table 1, C6M1 in water showed a combination of helical structure (37%) and random coil (45%) in its secondary structure. Introducing small amount of siRNA (MR of 40∶1) increased the absolute values in spectrum minima at 208 and 222 nm, and the maximum around 190 nm, which represent the helical structure. The helical content of C6M1 secondary structure increased to 81% at higher concentration of siRNA (MR of 10∶1). The secondary structure of C6M1 did not change by introducing more peptides, indicating a saturation point at MR of 10∶1. Considering the arrangement of amino acids in C6M1 (Figure 1), the ionic interaction between siRNA and arginine residues may stabilize C6M1 helical structure by neutralizing positive charge of arginine residues and reducing the charge repulsion between them. In HBS, however, the MRs of 20∶1 and 40∶1 showed the highest helical contents (69%) (Figure 6B and Table 1). Interestingly at MR of 10∶1, the presence of high amount of oligonucleotide and chloride anions led to a deformation of the CD spectra with a decrease in helical structure. This might be related to helix aggregation at high anion and RNA concentrations as also reported for CADY peptide [31].

**Table 1:** Secondary structure composition of C6M1 at different conditions.

Sample	α-helix (%)	r.c. (%)	Other (%)
C6M1 in water	37	45	18
MR = 40 in water	54	36	10
MR = 20 in Water	74	24	2
MR = 10 in Water	81	19	0
C6M1 in HBS	63	31	6
MR = 40 in HBS	69	27	4
MR = 20 in HBS	69	27	4
MR = 10 in HBS	26	50	24

r.c.  =  random coil; MR =  peptide:siRNA molar ratio; HBS =  HEPES-buffered saline.

### Agarose gel shift assay to characterize the interaction of C6M1 with siRNA and stability of the complex

Agarose gel shift assay was applied to evaluate the interaction between siRNA and C6M1 molecules and the stability of the formed complex in the presence of heparin and serum. Positively-charged peptides interact with siRNA mainly electrostatic interaction between basic residues and phosphate groups in siRNA backbone. Free negatively-charged siRNA molecules could move toward the positive electrode when the voltage is applied; while, stable peptide-siRNA complexes prevent the internalization of siRNA molecules into agarose gel, suggesting that there is no free siRNA to appear in siRNA bands.

**Figure 7:**
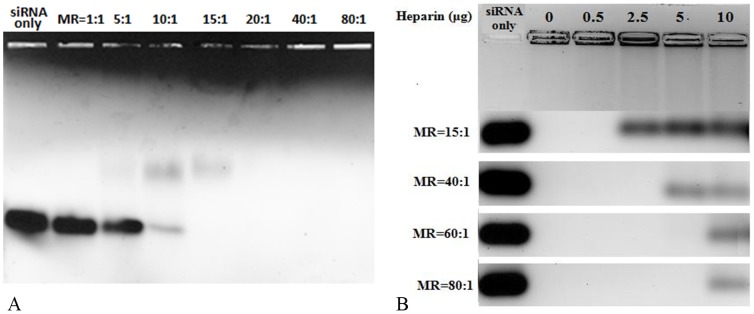
A) The formation of siRNA-C6M1 complex indicated by agarose gel, B) The stability of C6M1-siRNA complex indicated by heparin competition assay. Different amounts of heparin corresponding to final concentrations of 0.5 to 10 µg heparin per 10 µl of complex were added to C6M1-siRNA complexes at different molar ratios. The stability of complexes were analyzed by electrophoresis on agarose gel (1.2% wt/vol) stained with ethidium bromide. For better comparison, the siRNA bands of four independent gels were put in the same image.

Figure 7A shows the agarose gel shift assay of C6M1-siRNA complexes at different MRs. As shown, at MR of 1∶1, there was no significant difference between siRNA bands of free “siRNA only” and “MR = 1∶1” samples, suggesting that this MR was not enough to encapsulate the majority of siRNA molecules. The siRNA band in MR of 5∶1 was less bright than that of “siRNA only” sample, implying an effective interaction between C6M1 and siRNA molecules at this ratio. At the molar ratio of 10∶1, very small amount of free siRNA was observed on siRNA band, indicating that siRNA molecules were almost completely complexed with C6M1. The neutrally or positively charged complexes at this MR or higher were stuck in the wells and were unable to internalize the gel and move towards positive electrode, causing the darkness of the top of the gel. The siRNA band completely disappeared at the MR of 15∶1. Considering 7 arginine residues in C6M1 and 42 nucleotides in an siRNA molecule, 6 molecules of C6M1 should be theoretically enough to encapsulate one siRNA molecule; however, this finding suggests that excess C6M1 molecules are needed to achieve stable complexes.

Gel electrophoresis was also applied to study the stability of C6M1-siRNA complexes at different MRs in the presence of heparin. Heparin is an anionic competitive binding agent and a chemical analog of heparin sulphate proteoglycans (HSPG). The complex is expected to be stable at low concentration of heparin, as HSPG are abundantly found in the extracellular matrix and can dissociate the complex in extracellular environment. On the other hand, the complex should be able to dissociate and release siRNA easily, following cellular entry. As shown in Figure 7B, C6-siRNA complexes were stable in the absence of heparin (second well from left) and no free siRNA was shown in siRNA bands at all MRs. The complex at MR of 15∶1 was dissociated at heparin concentration of 2.5 µg per 10 µl of sample and higher. The minimum concentration of heparin required for dissociation of the complex increased by increasing the MR, indicating that higher amount of peptide could protect siRNA against dissociation from the complex. Interestingly, the complex at molar ratio of as high as 80∶1 released siRNA at high concentration of heparin, implying its willingness to release siRNA even at high MR.

### Stability of the complex to serum RNase degradation

Naked siRNAs are vulnerable to RNase degradation. In our study, we were interested in measuring the protection afforded by the peptide against serum RNase. Naked siRNA and C6M1-siRNA complexes at MR of 30∶1 were incubated in the presence of 50% active fetal bovin serum (FBS) and aliquots were taken at determined time intervals. Heparin was added to the complex after incubation with serum to release siRNA from the serum associated complexes.

**Figure 8:**
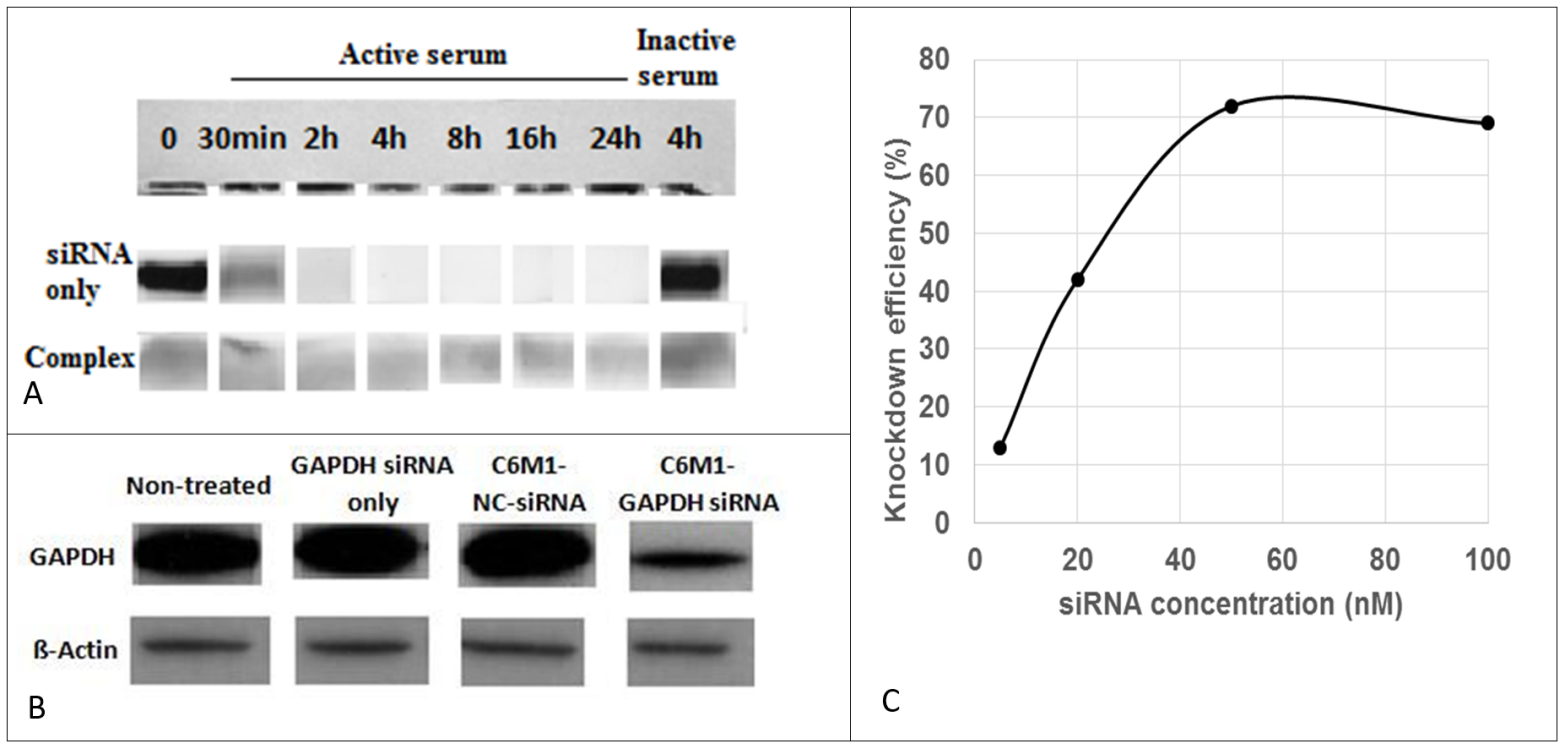
A) Stability of the C6M1-siRNA complexes to serum RNase degradation over time. Naked siRNA (top) or C6M1-siRNA complex (bottom) at molar ratio of 30∶1 were incubated in the presence of 50% active serum (FBS) over the period of 24 h or 50% heat-inactive serum (control) for 4 h. Aliquots (20 µl) were taken at 30 min, 2 h, 4 h, 6 h, 18 h and 24 h and EDTA (1 µl) was immediately added to stop the degradation. After the addition of 1% heparin to displace siRNA from the complex (bottom panel), aliquots were analyzed by 0.8% agarose gel electrophoresis. For better comparison, the siRNA bands of three independent gels were put in the same image. B) GAPDH protein levels, determined by western blotting, in the cells treated with C6M1-siRNA complexes or controls. CHO-K1 cells were treated with naked GAPDH siRNA or the complex of C6M1 with GAPDH siRNA (or negative control NC-siRNA) at MR of 30∶1 at 50 nM siRNA final concentration. 24 h post-treatment, the cells were lysed and analyzed by western blotting for the GAPDH protein levels as described in “Materials and Methods” section. β-actin was used as a control for quantification. C) Concentration dependent knock-down efficiency of C6M1-siRNA complexes at MR = 30∶1 and different concentration of siRNA. Gel images were analyzed by ImageJ software to quantify knock-down efficiency

As shown in Figure 8A, Naked siRNA (top panel) was completely degraded after 4 h incubation with active serum; however, it was stable in the presence of inactive serum. In contrast, C6M1 was able to protect siRNA even after 24 h incubation with high concentration of serum, showing the ability of C6M1 in protecting siRNA against serum RNase. The bands in the bottom panel are not sharp because of the presence of free peptide in the sample after dissociation by heparin. However, comparing top and bottom gel, for example, for 8 hr samples shows the presence of preserved siRNA in the bottom panel (complex).

### Knock-down efficiency of C6M1-siRNA complexes

The efficiency of C6M1 in intracellular delivery of siRNA and the knock-down of GAPDH gene were analyzed in protein level by western blotting technique. As shown in Figure 8B, the treatment of CHO-K1 cells with naked GAPDH siRNA did not change the level of this protein, implying that siRNA without an efficient carrier was not able to gain access to intracellular environment. However, the C6M1-siRNA complexes at siRNA concentration of 50 nM and MR of 30∶1 significantly decreased the level of GAPDH protein. Analysis of the gel images by ImageJ software showed ∼72% decrease in the GAPDH protein level in the cells treated with C6M1-GAPDH siRNA complexes compared to non-treated cells; while, those treated with naked siRNA or C6M1-NC siRNA showed no significant knockdown. β-actin protein was used in this experiment as an internal control for quantification. A concentration dependent study was also performed to identify the optimum siRNA concentration for *in vitro* transfection experiments. As shown in Figure 8C, the best siRNA concentration to achieve efficient knock-down was 40-50 nM.

## Conclusions

Understanding the properties of peptides is necessary for their effective use as siRNA delivery systems. C6M1, an 18-mer amphipathic peptide, formed small complexes in water and HEPES (<200 nm), but aggregated to larger particles in PBS. Using DLS and fluorescence spectroscopy, the study of the aggregation kinetics of complex in PBS revealed that the size of the complex increased at the first 1 h incubation but remained almost constant afterwards. The secondary structure of C6M1 in water involved a combination of helical and random coil structures; however, upon binding to siRNA or in the presence of anions, C6M1 adopted mainly an α-helical structure. Agarose gel experiments showed the ability of C6M1 to completely encapsulate siRNA molecules at molar ratio of 15∶1; however, higher molar ratios were required to achieve stable complexes in PBS. C6M1 showed high capability in protecting siRNA against serum nuclease over the period of 24 h, while naked siRNA was completely degraded in 4 h. Western blotting experiment showed ∼72% decrease in GAPDH protein content of the cells treated with C6M1-siRNA complexes. Taking all the results into account, C6M1 demonstrated potential as a safe carrier for siRNA delivery. The importance of choosing an appropriate medium to control the peptide secondary structure and complex size was also highlighted in this study.
